# Adherence to Supervised and Unsupervised Exercise Programmes in Ageing Population with Intermittent Claudication: A Randomized Controlled Trial

**DOI:** 10.3390/jcm13133690

**Published:** 2024-06-25

**Authors:** Cecilia Herrero-Alonso, Victor-Miguel López-Lifante, Anna Costa-Garrido, Guillem Pera, Maite Alzamora, Rosa Forés, Esau José Martínez-Ruíz, Juan López-Palencia, Luciana Moizé-Arcone, Ester Mateo-Aguilar, Vanesa Rodríguez-Sales, Marina Alventosa, Antonio Heras, Marta Valverde, Concepció Violán, Pere Torán-Monserrat

**Affiliations:** 1 Unitat de Suport a la Recerca Metropolitana Nord, Institut Universitari d’Investigació en Atenció Primària Jordi Gol (IDIAP Jordi Gol), 08303 Mataro, Spain; cherreroal.mn.ics@gencat.cat (C.H.-A.); vmlopezli.mn.ics@gencat.cat (V.-M.L.-L.); annacostaga@gmail.com (A.C.-G.); gpera@idiapjgol.info (G.P.); maiteal2007@gmail.com (M.A.); rosafores2011@gmail.com (R.F.); malventosa.bcn.ics@gencat.cat (M.A.); aheras.bnm.ics@gencat.cat (A.H.); mvalverde@hospitalmeritxell.ad (M.V.); ptoran.bnm.ics@gencat.cat (P.T.-M.); 2 Palau-Solità Primary Healthcare Institut Català de la Salut, Centre Palau-Solità Plegamans, 08184 Barcelona, Spain; 3 Department of Medicine, Universitat Autònoma de Barcelona, 08193 Bellaterra, Spain; 4 Multidisciplinary Research Group in Health and Society (GREMSAS), Institut Universitari d’Investigació en Atenció Primària Jordi Gol (IDIAPJGol), 08007 Barcelona, Spain; 5 Vascular Surgery Service, Hospital de Mataró, 08916 Barcelona, Spain; emartinezru@csdm.cat (E.J.M.-R.); jlopalencia@gmail.com (J.L.-P.); 6 Research Group in Technology Applied to High Performance and Health, Department of Health Sciences, TecnoCampus, Universitat Pompeu Fabra, Av. d’Ernest Lluch, 32, 08302 Barcelona, Spain; lmoize@tecnocampus.cat (L.M.-A.); rodriguezsalesv@gmail.com (V.R.-S.); 7 Research Group GRACIS, Department of Health Sciences, TecnoCampus, Universitat Pompeu Fabra, Av. d’Ernest Lluch, 32, 08302 Barcelona, Spain; emateo@tecnocampus.com; 8 Emergency Department, Hospital Nostra Senyora de Meritxell, AD700 Escaldes-Engordany, Andorra; 9 Grup de Recerca en Impacte de les Malalties Cròniques i les Seves Trajectòries (GRIMTra), Institut Universitari d’Investigació en Atenció Primària Jordi Gol (IDIAPJGol), 08303 Barcelona, Spain; 10 Germans Trias i Pujol Research Institute (IGTP), 08916 Badalona, Spain; 11 Red de Investigación en Cronicidad, Atención Primaria y Prevención y Promoción de la Salud (RICAPPS), Instituto de Salud Carlos III (ISCIII), 28029 Madrid, Spain; 12 Department of Medicine, Faculty of Medicine, Universitat de Girona, 17001 Girona, Spain

**Keywords:** clinical trial, intermittent claudication, adherence, medical education and training, primary health care, vascular medicine, sports medicine

## Abstract

**Background**: Intermittent Claudication symptomatic peripheral arterial disease (ICSPAD) is associated with reduced mobility, functional capacity, and quality of life. Physical exercise is an effective non-pharmacological intervention for the management of ICSPAD. Adherence to exercise programs is challenging, due to the nature of the disease and the complex comorbidities associated with it. This study aimed to determine adherence to three supervised physical exercise programs (a walking intervention, strength intervention, and concurrent intervention) and an unsupervised exercise program (standard advice) in individuals with ICSPAD. **Methods**: In this clinical trial, 122 patients were divided into four groups based on the type of exercise program they followed: standard advice, walking intervention, strength intervention, and concurrent intervention. **Results**: The results revealed that while the demographic characteristics were similar, the strength intervention group had a younger mean age, and the walking group had a higher prevalence of hypertension and increased usage of anti-hypertensive drugs. Adherence to physical exercise and pedometer wearing was highest in the standard advice group. Logistic regression analysis showed lower odds of adherence to exercise and pedometer wearing in the intervention groups compared to the standard advice group. Adherence did not significantly vary across ankle-brachial index categories. Furthermore, there was no significant difference in adherence between the severity levels of intermittent claudication, though mild cases tended to exhibit higher adherence. **Conclusions**: The results show that the standard advice from healthcare professionals positively influences treatment adherence.

## 1. Introduction

Patients afflicted with peripheral arterial disease (PAD) exhibit diminished arterial perfusion in the lower limbs, primarily due to atherosclerotic plaque-induced luminal narrowing, restricting distal blood flow and causing intermittent claudication—pain in the thigh or calf during exertion, due to temporary leg muscle ischemia [1]. Although some PAD patients are asymptomatic or exhibit atypical symptoms, diagnosing PAD remains crucial due to its association with systemic atherosclerosis, carrying a cardiovascular risk equivalent to previous myocardial infarction. Risk factor modification is necessary for PAD patients to enhance long-term survival, with management strategies including lifestyle adjustments, risk factor reduction, pharmacotherapy, endovascular intervention, and surgery, tailored to disease severity and symptomatology [2]. PAD affects over 236 million people worldwide and it is projected to increase by 40% in the coming years [3].

Major risk factors for PAD include age, smoking, diabetes, and dyslipidemia. The most common symptom of PAD is intermittent claudication (IC), present in 40 to 75% of cases [1]. IC is a common symptom experienced by individuals with PAD. It is characterized by exercise-induced lower limb pain or cramping that is relieved by rest [4]. The muscle ischemia precipitated by exercise is frequently associated with reduced walking capabilities. Consequently, these patients have lower physical activity levels compared to their age-matched healthy controls [5,6].

People with IC are 3–6 times more likely to experience a cardiovascular event (stroke, angina, heart attack, or death) [4].

Supervised exercise programs (SEP) involve structured sessions conducted in a controlled setting, often under the guidance of healthcare professionals or exercise specialists. These programs typically consist of aerobic exercises, such as walking or cycling, combined with strength training. SEP provides a supportive environment for patients, allowing close monitoring of their progress and adjustments to the exercise prescription as needed.

Unsupervised exercise programs (USEP) offer an alternative approach, providing patients with the flexibility to perform exercises in the comfort of their own home. These programs often include walking regimens, supported by educational materials and remote monitoring to ensure proper adherence and progression. Home-based exercise aims to empower patients to actively manage their condition and integrate exercise into their daily routines.

Combination exercise programs incorporate both aerobic and resistance training components. Aerobic exercises improve cardiovascular fitness, while resistance exercises enhance muscle strength and endurance. Combining these exercise modalities may offer synergistic benefits, addressing multiple aspects of IC management simultaneously.

Despite the demonstrated effectiveness of exercise programs in improving IC symptoms and functional outcomes, adherence to these interventions remains a challenge. Adherence refers to the extent to which individuals consistently engage in and comply with prescribed exercise regimens. Factors influencing adherence include motivation, social support, perceived barriers, and individual preferences. Understanding and addressing these factors is essential for optimizing adherence rates and maximizing the benefits of exercise interventions for IC patients.

Although there is evidence that physical exercise, whether supervised or not, improves IC, walking capacity, and the day-to-day lives of our patients with symptomatic PAD disease, they do not consistently and progressively engage in it [7].

There are several reasons why people with IC may not engage in regular and consistent exercise, including pain and discomfort, lack of motivation, physical barriers, economic barriers, lack of knowledge about the benefits of physical exercise, and coexisting health problems such as heart or respiratory disease [8,9,10,11].

Further research is needed to identify effective strategies for promoting and sustaining adherence to exercise programs in IC patients, ultimately optimizing their outcomes and quality of life. Thus, this study aimed to determine the level of adherence to three supervised physical exercise programs (walking intervention, strength intervention, concurrent intervention) and an unsupervised exercise program (standard advice) in patients over 40 years with IC in the Northern Metropolitan Area of Barcelona (Spain).

## 2. Materials and Methods

### 2.1. Design, Setting, and Study Population

A four-group parallel, longitudinal, randomized controlled trial, blind to analysis, with a sample of participants studied for between 3, 6, and 12 months after starting a physical exercise program was carried out in the Maresme Region, Catalonia (Spain), a Mediterranean region of 459,625 inhabitants.

Inclusion criteria were as follows: (1) patients with PAD who had visited the vascular surgery service at Mataró Hospital, had an ankle-brachial index (ABI) of less than 0.9, and IC; (2) aged 40 years or older; and (3) had read the patient information sheet, understood, and signed informed consent.

The exclusion criteria were as follows: (1) critical ischemia and/or acute ischemia of the lower limbs; (2) previous bilateral revascularization of the lower limbs; (3) lower limb amputation, (4) cardiovascular, pulmonary, neurological, and osteoarticular diseases that prevent the performance of the intervention; (5) appearance of IC beyond 30–45 min after the start of claudicometry; and (6) refusal of the patient to participate in the study, inability to go to the center due to lack of time, or limiting disease.

### 2.2. Participant Recruitment, Follow-Up

**Figure 1:**
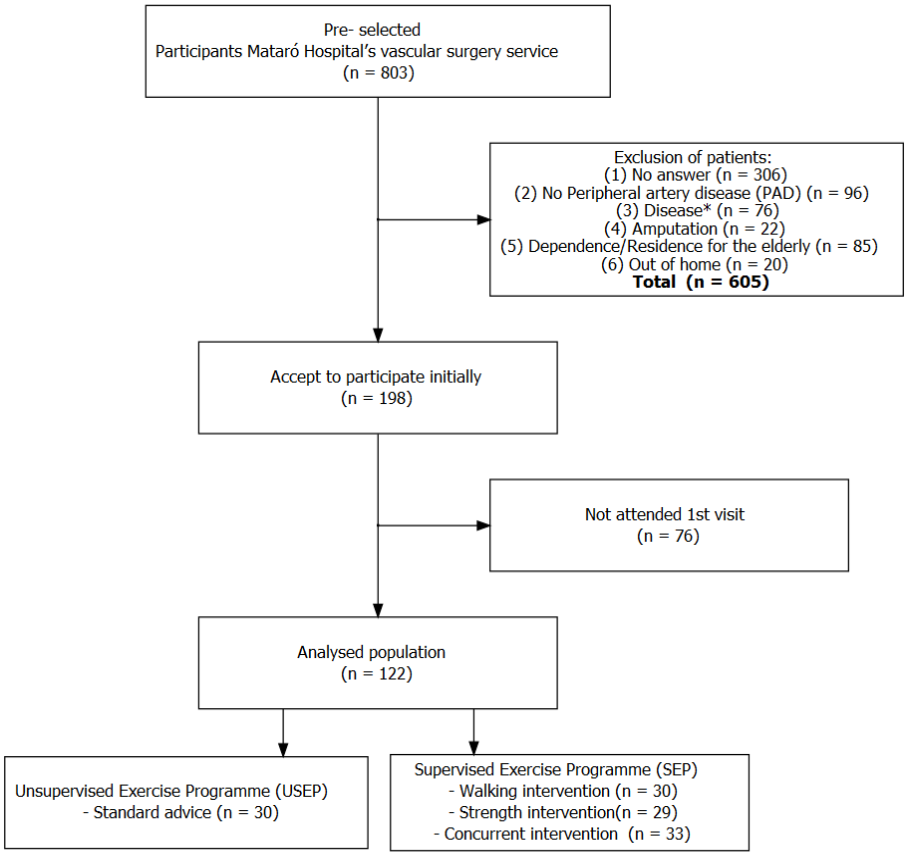
Flowchart of the study population. The figure reports the number of individuals who met each exclusion criterion, as well as the number of individuals that met all the criteria. * Cardiovascular, pulmonary, neurological, and osteoarticular diseases that prevented performance of the intervention.

Participants were recruited in the outpatient clinics of the Vascular Surgery Service of Mataró Hospital in a consecutive manner, being called from the list of patients seen between 3 September 2021 and 15 September 2022. Participants (*n* = 76) who did not attend the clinic at visit 1 and were therefore not randomized were considered dropouts (Figure 1). At baseline, they underwent an eligibility assessment based on the inclusion criteria, with no inclusion or exclusion criteria. Eligible participants were then invited to participate and, if accepted, underwent inclusion visits, randomization, intervention, and follow-up assessments at a later date. There were 3 visits: visit 0 (eligibility assessment), visit 1 (baseline), and visit 2 (intervention assessment after 3 months). At each visit, the following assessments were made:

Visit 0. Assessment for eligibility. ABI and claudicometry (performed by vascular surgery), and sign informed consent.

Visit 1. Baseline: Collection of sociodemographic data, cardiovascular risk factors, previous vascular disease, drugs, treadmill walk distance, 6 min walk distance, life quality questionnaires, and functional status questionnaires. The 122 patients were randomized into 4 groups: 3 groups were intervention groups (WI, SI, CI), with a control group (SA) (Figure 1).

Visit 2: Assessment of sociodemographic data, cardiovascular risk factors, previous vascular diseases, ABI, treadmill based on walking/cardiorespiratory fitness assessment, 6 min walk distance, muscle fitness assessment, perceived quality of life level through the specific questionnaires for PAD (VascuQOL-6), and quality of life questionnaires (SF-12) [12]. VascuQOL-6 is a validated tool that measures the impact of vascular conditions on various aspects of a patient’s life, providing valuable insights into their well-being and functioning. The Barthel scale was also assessed. This is an ordinal scale used to measure a person’s ability to perform activities of daily living (ADL) [13].

### 2.3. Randomization

After patients agreed to participate in the study, the statistician assigned each patient to either the control group or one of the three intervention arms. An Excel sheet was utilized, containing columns for “group”, “age group (in 5-year increments)”, “gender”, and “severity of intermittent claudication (using the Rutherford questionnaire)” (mild IC > 300 m; moderate IC: 100–300 m; severe IC < 100 m) [14]. The rows were randomly sorted. The statistician then selected the first unassigned row that matched the selected participant. Participants were assigned to the control group (standard advice) and the three intervention groups (walking intervention, strength intervention, concurrent intervention) through randomization.

### 2.4. Intervention Programs and Control Group on Physical Exercise

*Walking intervention (WI) group*: The intervention involved conducting a total of 36 sessions of treadmill training, with exactly three sessions per week for 3 months, each lasting 60 min. The initial determination of the comfortable walking speed and slope degree was based on data obtained during Visit 1 (session 1), specifically focusing on treadmill-based walking and the 6 min walking distance. These parameters were adjusted for each patient until symptoms of moderate intermittent claudication (grade 3–4 per the claudication symptom rating scale) manifested after 5–10 min of activity. Subsequently, a period of rest (either sitting or standing) was observed until the lower limb pain subsided. This cycle continued until the completion of the 60 min session duration established for each patient. Over time, the intensity of speed and slope inclination was progressively increased, tailored individually for each patient’s tolerance and progression.

From session 2 through session 36, the exercise program established in session 1 was consistently maintained until the completion of the 36 sessions.

*Strength intervention (SI) group*: The training regimen comprised a resistance exercise program for 36 weeks, featuring three 60 min weekly sessions dedicated to exercises involving resistance. These exercises followed similar guidelines as the walking regimen but focused on applying strength stimuli. Should symptoms of moderate intermittent claudication (rated 3–4 on the claudication symptom rating scale) arise within 5–10 min, participants were instructed to rest (either sitting or standing) until the lower extremity pain subsided. This cycle was repeated until the conclusion of each strength training session. The progression of strength stimuli was adjusted weekly based on the patients’ subjective perception of effort.

*Concurrent intervention (CI) group:* A training program was implemented involving alternating strength and endurance stimuli during the same session for 36 weeks. Each week, participants engaged in three 60 min sessions, with 35 min dedicated to resistance exercises. To complete the remaining 25 min, guidelines akin to those used in the walking exercise were followed, applying resistance stimuli. Should symptoms of moderate intermittent claudication at grade 3–4, as per the claudication symptom rating scale, manifest within 5–10 min, participants were instructed to rest (sitting or standing) until the lower limb pain abated. This pattern was repeated until the designated 20 min mark for the conclusion of the concurrent training session was reached. Weekly increments in the intensity of strength and endurance stimuli were made based on the subjective perception of exertion by the patients.

All physical activity interventions (WI, SI, and CI) were performed by physiotherapists and nurses. They were carried out at the Department of Health Sciences, TecnocaCampus, Universitat Pompeu Fabra (UPF), located 6 km from Mataró Hospital and accessible by public transport.

*The standard advice (SA) group* was a control group in which the patients received standard advice from a healthcare provider, recommending performing aerobic exercises at the lower limb level. Participants were instructed to cease exercise upon the onset of pain, resuming only when the pain diminished, and to complete daily 30 min sessions of exercise over 3 months at home without supervision.

In addition to intervention or standard advice programs, participants in each group were instructed to consistently utilize a pedometer from Visit 1 to Visit 2 over three months. The daily step count was considered if they took more than 10 steps.

### 2.5. Main Outcomes

Adherence to physical exercise: Completion of a minimum of 75% of sessions was required. Specifically, participants in the WI, SI, and CI groups were expected to complete at least 27 out of 36 sessions. In contrast, participants in the SA group were required to complete at least 65 or 69 days of aerobic exercise at home, depending on whether the three-month period comprised 87 or 92 days, respectively.

Adherence to wearing a pedometer: Attainment of at least 75% compliance in wearing a pedometer was required. This ratio was calculated by considering the number of days with ≥10 steps recorded relative to the total number of days within the three months.

### 2.6. Outcome Measures

Sociodemographics: age and gender (male, female).

Cardiovascular risk factors: smoking [smoker (cigarettes/day, packs/year), ex-smoker, never smoker]; hypertension; diabetes; hypercholesterolemia; obesity (body mass index ≥ 30 kg/m^2^).

Previous cardiovascular disease: ischemic heart disease (angina, acute myocardial infarction, coronary revascularization); cerebrovascular disease (transient cerebral vascular accident, stroke, cerebral revascularization); symptomatic abdominal aortic aneurysm; other revascularizations (aorto-femoral bypass).

Drugs that can modify the clinical course of IC such as anticoagulants, hypotensive, lipid-lowering, and hypoglycaemic drugs during the study period. Cilostazol, antiplatelet agents, and non-steroidal anti-inflammatory drugs (NSAIDs).

Ankle-brachial index (ABI): Ratio of the higher of the two systolic pressures (tibial posterior and dorsal artery) and arm systolic pressure using the systolic pressure of the highest arm Mild ischemia: 0.89–0.71; Moderate ischemia 0.70–0.51; Severe ischemia: ≤0.50.

Claudication walk test (CWT): The distance walked in meters until the onset of pain, as measured by the Rutherford questionnaire, served as an indicator of the severity of intermittent claudication (mild IC > 300 m; moderate IC: 100–300 m; severe IC < 100 m).

Barthel questionnaire: Consists of 10 items that measure a person’s daily functioning. (total dependence < 20; severe dependence 20–935; moderate dependence 40–955; mild dependence 60–99; independence = 100).

SF-12 questionnaire: A self-reported outcome used as a quality of life measure. It comprises physical and mental component scores. Both are numerical ordinal variables ranging from 0 to 100.

VascQol-6 questionnaire: A numerical ordinal variable ranging from 4 to 24 that is utilized as a valid and responsive instrument for assessing health-related quality of life in PAD.

See previous publication for more details on the study protocol [15].

### 2.7. Sample Size

A sample of 124 patients, 31 per group, was needed to detect a 155 m improvement in pain-free walking distance after the intervention, assuming that the SD of the improvement was also 155 m. This is similar to an increase of 159s walking without pain, for an average speed of 3.5 km/h, as has been described previously [16]. This calculation is comparable to any improvement as long as the SD was equal to or less than this improvement and to comparisons between the control group and any of the other groups (correction for three comparisons using Bonferroni). This calculation is analogous to the rest of the result variables (Barthel, SF-12, VascQOL-6, ABI) as long as the difference observed between groups is equal to or greater than the common SD (primary outcome 2). The correlation between baseline and final measurement was assumed to be 0.5. For comparisons between the treatment and control groups using the proportional version (proportion of patients improving in one group vs. the other), the differences should be 50% improvement vs. 15% or, equivalently, 35% vs. 5%. It was assumed that the correlation between baseline and final intervention measurements was 0.5, with a 20% follow-up loss rate, an alpha risk of 0.05, 80% power, and two-sided tests.

### 2.8. Statistical Analysis

Descriptive analyses were conducted to characterize each of the intervention programs and control group, and assess the relationship between adherence to physical exercise and ABI, as well as the severity of IC. Categorical variables were assessed using frequencies and percentages, while continuous variables were analyzed using means with standard deviations (SD) or medians with interquartile range (IQR) in cases where normality assumptions were not met. Group comparisons for percentages involved $\chi^{2}$ tests when cell counts were higher than 5; otherwise, the Fisher exact test was employed. Analysis of variance was utilized for means, and the Kruskall–Wallis test was applied for medians across all four groups. Adherence to physical exercise was compared across the control group and the other three interventions using logistic regression. The analysis incorporated adjustments for age, various aspects of disease history (hypertension, diabetes, global obesity with body mass index (BMI) ≥ 30), medication use (anti-hypertensive, antidiabetic, hypolipidemic), and VASCUQOL-6. Similarly, logistic regression was applied to assess adherence to wearing a pedometer within the control group and the three other interventions.

All tests were two-sided, and a statistical probability of p<0.05 was considered significant. All analyses were performed using R version 4.2.2.

### 2.9. Ethics

The ethics committees of the Foundation University Institute for Primary Health Care Research Jordi Gol i Gurina (IDIAPJGol) (ref. 20/035-P) approved the study protocol. All participants recruited to the study were fully informed about the study protocol and signed an informed consent to participate. They consented to use their collected data for research and agreed to the applicable regulations, privacy policies, and terms of use. Participant data weren anonymized according to a numerical coding system based on their order and securely stored in a database (REDCap). The database will be maintained for a period of 15 years after the completion of the study. No participants or members of the public were directly involved in the design or analysis of the reported data. The funders of the study had no role in the study design, data collection, data analysis, data interpretation, or writing of the report. The corresponding author (CV) had full access to all data, while AC and GP had access to the raw data. Trial registration number NCT04578990.

## 3. Results

### 3.1. Study Population Characteristics

We analyzed 122 patients with intermittent claudication who participated in the study and had similar characteristics. However, in the intervention group SI, the mean age was lower compared to the other groups (*p* < 0.005). No differences were observed in gender distribution among the three intervention programs and standard advice. Notably, most subjects were men, with no significant differences in gender distribution observed among the three intervention programs and the standard advice group. Analysis of disease history revealed a significantly higher proportion of subjects with hypertension in the WI group compared to the others, while no statistically significant distinctions were identified for the prevalence of diabetes, obesity, or hypercholesterolemia. Furthermore, the proportions of comorbidities such as myocardial infarction, angina, stroke, and transient ischemic attack were similar across all participants. Concerning drug utilization, a significantly higher proportion of subjects in the WI and SA groups were found to be using anti-hypertensive medication compared to the SI and CI groups. Additionally, the WI group exhibited a higher proportion of subjects using antidiabetic medication compared to the other three, although this difference did not reach statistical significance. These differences corresponded to the percentage of hypertensive and diabetic individuals in each group (see Table 1).

### 3.2. Adherence to Physical Exercise Programs

Comparison of the different physical exercise intervention programs was carried out using four parameters (measures): ratio of adherence to physical exercise, adherence to physical exercise ≥75%, ratio of adherence to wearing a pedometer, and adherence to wearing a pedometer ≥75%. Notably, USEP demonstrated the highest adherence to both physical exercise and steps, while those in the SEP group exhibited the lowest adherence overall (see Table 2).

**Figure 2:**
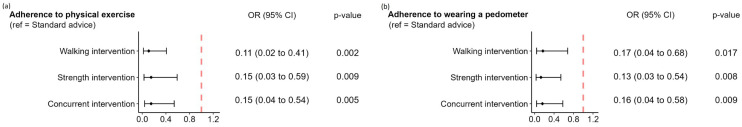
Logistic regression models assessing (**a**) the adherence to physical exercise and (**b**) the adherence to wearing a pedometer between the intervention and standard advice groups. Note: All multivariate analysis were adjusted for age, different aspects of the disease history (hypertension, diabetes, global obesity BMC ≥ 30), drugs (anti-hypertensive, antidiabetic, hypolipidemic), and VASCUQOL-6. Abbreviations: OR, odds ratio; CI, confidence interval.

Figure 2 illustrates the findings from a logistic regression analysis exploring the adherence to physical exercise programs between the interventions and control group, while accounting for demographic and health-related factors. All three interventions exhibited significantly lower odds of adherence to physical exercise compared to the SA, with ORs ranging from 0.11 to 0.15 and *p*-values between 0.002 and 0.009. Similarly, for adherence to wearing a pedometer, the three intervention groups demonstrated significantly lower odds of adherence compared to the SA, with ORs ranging from 0.13 to 0.17 and *p*-values between 0.008 and 0.017. Additionally, age, various aspects of disease history, drugs, and VASCUQOL-6 were adjusted for, but none were significantly associated with adherence (*p*-value > 0.05).

The adherence to physical exercise of at least 50% and the wearing of a pedometer were also examined (see Table 3). Similarly to in Table 2, USEP demonstrated the highest adherence to both physical exercise and steps, whereas those in the SEP group exhibited the lowest overall adherence. Nevertheless, it was also noted that WI and CI showed compliance rates to physical exercise exceeding 60%.

### 3.3. Adherence to Physical Exercise and Ankle-Brachial Index

Table 4 presents the count and proportion of participants in each ABI category who adhered to the physical exercise program, categorized by intervention programs and the control group. The overall adherence did not exhibit significant variation across the ABI categories (*p* = 0.210). Likewise, there were no notable differences in adherence rates among the ABI categories within the SA (*p* = 0.274), WI (*p* = 0.550), SI (*p* = 0.537), or CI (*p* = 0.285) groups.

### 3.4. Adherence to Physical Exercise and Claudication Walk Test

Table 5 shows the relationship between adherence to physical exercise and the severity of IC as assessed by the CWT. Patients were categorized into severe and moderate IC (CWT score of 300 or less) or mild IC (CWT score greater than 300), and the table displays the count and proportion of adherent patients in each group, stratified by intervention type and control group. The findings indicate no statistically significant difference in adherence to physical exercise between patients with severe/moderate IC and those with mild IC (*p* = 0.154). Notably, there is a trend suggesting higher adherence in the mild IC group. Additionally, the table illustrates variability in adherence across different intervention types, but the consistency of the relationship between adherence and severity of IC is reflected by non-significant *p*-values for each intervention type and control group.

## 4. Discussion

Our study was based on the assessment of adherence to different types of physical activity in a representative and well-characterized sample of patients over 40 years of age with ICSPAD seen at the Vascular Surgery Service and Primary Health Care Centres of Mataró Hospital and the School of Health Sciences, TecnoCampus. Adherence to exercise intervention programs was low. Adherence was not influenced by sociodemographic characteristics or associated health factors, nor by the severity of intermittent claudication or ankle-brachial index level.

In this study, the adherence threshold was considered to be attendance at 75% of the physical exercise sessions. The same level of adherence was considered for wearing the pedometer. This threshold varied between the different studies reviewed [10,17,18]. Research indicates that adherence rates to supervised exercise therapy (SET) for peripheral artery disease (PAD) vary, with an average completion rate of 75.1% among participants who started a SET program [10]. Nevertheless, in another study focusing on a 12-week PAD-focused SET program, the overall adherence was reported at 62%, with differences observed based on the exercise mode used [17]. The study found that a multimodal approach involving recumbent total body stepping (step-ex) had higher adherence rates compared to treadmill walking alone, suggesting the potential benefits of incorporating different exercise modes to improve adherence [17]. We did not observe that concurrent exercise rates were higher than those of WI or SI. In this study, if we consider adherence at the 50% threshold, we observe that the WI and CI exhibited compliance rates exceeding 60%, suggesting that these types of patients were not adherent to follow-ups longer than 6–7 weeks, an insufficient timeframe to observe improvements in ICSPAD [17].

The supervised walking intervention and the concurrent intervention programs showed the highest adherence. However, this adherence was no better than standard advice. In this intervention, patients performed exercises at home or on behalf of patients. These results could be explained by the fact that certain prognostic variables of exercise adherence were not taken into account in the study. For example, regarding patient-related factors such as prior exercise habits, motivation, and perceived behavioral control, which could influence the outcomes [19]. However, socioeconomic aspects, medication, and associated comorbidity were considered, with no differences observed between the physical intervention groups and standard advice. Importantly, the study results could also have been influenced by the low medication adherence among patients with heart failure. Medication non-adherence is a known issue, with studies reporting adherence rates as low as 30–57% for cardiovascular preventive medications [20,21].

The adherence to non-supervised exercise in intermittent claudication is a critical aspect to consider. While supervised exercise therapy has shown significant benefits for patients with intermittent claudication, adherence to non-supervised exercise programs may face challenges [22]. One study indicated that approximately 30% of respondents in a European survey lacked access to supervised facilities, referral pathways, and trained staff, highlighting potential barriers to adherence to supervised programs [23]. Additionally, some patients may find supervised programs inconvenient due to financial, time, or public or private transport limitations, which could impact their willingness to participate consistently in such programs. Therefore, ensuring the availability of alternative home-based or self-facilitated exercise programs is essential to improve adherence rates and provide options for patients who may face obstacles in accessing supervised exercise therapy [23].

Supervised exercise training has clinically meaningful benefits for walking ability and quality of life. Several national and international guidelines recommend walking, and it is the exercise modality with the strongest evidence [17]. Evidence for progressive resistance training is growing, and patients can also carry out strength training alongside a walking program. For people who are unable to attend a supervised exercise class (strongest evidence), home or ‘self-directed’ exercise programs have been shown to improve walking distance compared with simple advice. All exercise programs, regardless of delivery method, should be progressive and, where possible, individualized. They should take into account disease severity, comorbidities, and initial exercise capacity [17]. The walking intervention was adapted to the characteristics of each patient, the starting speed was not the same for each patient, unlike other protocols that suggested the same starting speed for all participants in the intervention [19]. Several studies demonstrated improvements in treadmill walking after 3 months of supervised exercise. However, given the characteristics of the study designs and patient features, it is likely that the optimal prescription would be difficult to elucidate due to the heterogeneity of the studies, including differences in exercise frequency, intensity, and type [24,25]. In this study, although there were no objective data, we believe that the role of health professionals, hospital and primary care, was crucial in standard advice.

Research indicates that factors such as characteristics of the exercise program, involvement of professionals from different disciplines, supervision, technology, social support, communication, feedback, self-efficacy, and goal setting play crucial roles in increasing adherence to physical exercise among older adults [26]. Additionally, the type of exercise, intensity, frequency, volume, and duration of the program impact adherence levels. For instance, alternative exercise options like Nordic walking or resistance training may improve adherence compared to traditional interventions like walking. Moreover, the frequency and duration of the exercise program can significantly affect adherence rates [18,22].

The strengths of this study are as follows: This study encompassed a broad array of supervised exercise programs in patients with CI. A wide variety of training regimens were included to enhance symptomatology and quality of life in these patients. The study assessed three different types of training plans. However, no significantly higher adherence was observed among any specific activity when adherence was considered to be superior to 75%. This threshold represents the high cut-off point necessary to detect differences that justify application in routine clinical practice. The fact that no significant differences were observed in adherence to the different programs suggests that there was a common cause for the low adherence. Two types of reasons could explain these results: intrinsic patient factors, such as prior experience with physical exercise; and extrinsic factors, such as the location of the activities, and the proximity or distance from the patient’s residence, which influence adherence rates.

Interpretation of these results should take into account the limitations of the study. First, we did not have information about participants who decided not to participate in the clinical trial, which could have introduced a selection bias. However, this bias was minimized, since the participants who chose not to participate had a similar clinical situation and were from the same geographical area. Second, we did not include certain prognostic factors of adherence to exercise like self-efficacy that are considered emerging factors for adherence to home exercise [19,27,28]. Studies led by Essery and Jack observed that self-efficacy was a prognostic factor for adherence [27,28]. They reported that individuals with higher self-efficacy were more likely to be more adherent to outpatient exercise. Higher self-efficacy and confidence in their ability to complete a given task allow patients to overcome challenges with greater ease, which may be particularly important in home settings where there is no professional supervision. Third, differences due to gender or IC severity were not considered in the analysis. In addition to these, the study could be extended to include many other factors such as age, nationality, socioeconomic status, or comorbidities, but the homogeneity of the sample in these aspects did not allow for sufficient statistical power to perform this. Exercise, even unsupervised and low-intensity exercise, should be recommended and encouraged for all patients with PAD and without contraindications. However, several factors, such as medical comorbidities, referral and insurance systems, and patient willingness to participate and adhere, limit the use of supervised exercise programs.

Future work could widen the recruitment of the sample to include more diverse individuals in these respects, to increase the generalizability of the findings. In addition, given that the intervention was not positive, it would be interesting in the future to consider the perceptions of both the patients and professionals who delivered the intervention.

## 5. Conclusions

To conclude, the supervised physical exercise programs were inferior to those of unsupervised physical activity. Adherence did not vary according to the severity of ICSPAD, gender, or comorbidities. Adherence to physical exercise programs in patients with intermittent claudication needs to be adapted and personalized for each individual to ensure proper adherence. This study in older people with intermittent claudication showed that unsupervised exercise achieved better adherence than supervised exercise, in contrast to other studies where supervised exercise resulted in better adherence. We emphasize how difficult it is to adhere to supervised physical exercise not performed at home or in a place close to home, especially in elderly patients with multiple pathologies. The results show that the standard advice given by healthcare professionals is a positive intervention that can achieve treatment adherence.

## Figures and Tables

**Table 1:** Basal characteristics of the subjects with intermittent claudication according to physical activity.

	Standard Advice (SA)		Walking Intervention (WI)		Strength Intervention (SI)		Concurrent Intervention (CI)			Total	
	n = 30		n = 30		n = 29		n = 33			n = 122	
	Mean/n	SD/%	Mean/n	SD/%	Mean/n	SD/%	Mean/n	SD/%	* p * Value	Mean/n	SD/%
Age (years)	69.07	7.12	69.93	6.58	64.14	5.99	67.97	6.14	**0.005**	67.81	6.75
Male	28	93.33	25	83.33	28	96.55	31	93.94	0.354	112	91.80
Smoking status									0.162		
Smoker	10	33.33	9	30.00	15	51.72	13	39.39		47	38.52
Never smoker	5	16.67	1	3.33	5	17.24	4	12.12		15	12.30
Ex-Smoker	15	50.00	20	66.67	9	31.03	16	48.48		60	49.18
**Disease** **history**											
Hypertension	22	73.33	28	93.33	19	65.52	20	60.61	**0.013**	89	72.95
Diabetes	16	53.33	23	76.67	13	44.83	16	48.48	0.058	68	55.74
Global obesity (BMI ≥ 30 kg/m^2^)	13	43.33	10	33.33	4	13.79	8	24.24	0.073	35	28.69
Hypercholesterolemia	24	80.00	26	86.67	23	79.31	29	87.88	0.751	102	83.61
**Comorbidities**											
Myocardial infarction	8	26.67	9	30.00	8	27.59	11	33.33	0.940	36	29.51
Angina	6	20.00	8	26.67	5	17.24	6	18.18	0.801	25	20.49
Stroke	3	10.00	5	16.67	3	10.34	3	9.09	0.833	14	11.48
Transient ischemic attack	2	6.67	1	3.33	0	0.00	1	3.03	0.743	4	3.28
Deep vein trombosis	3	10.00	1	3.33	1	3.45	0	0.00	0.217	5	4.10
Pulmonary embolism	1	3.33	1	3.33	0	0.00	0	0.00	0.602	2	1.64
Revascularization	13	43.33	14	46.67	17	58.62	16	48.48	0.676	60	49.18
Type of angioplasty											
Cardiac	7	23.33	7	23.33	8	27.59	6	18.18	0.854	28	22.95
Cerebral	1	3.33	0	0	0	0	2	6.06	0.617	3	2.46
Lower limbs	5	16.67	8	26.67	9	31.03	9	27.27	0.619	31	25.41
Abdominal aortic aneurysm	0	0.00	0	0.00	1	3.45	0	0.00	0.238	1	0.82
COPD	12	40.00	6	20.00	12	41.38	7	21.21	0.121	37	30.33
Asthma	1	3.33	0	0.00	2	6.90	1	3.03	0.550	4	3.28
**Drugs**											
Anti-hypertensive	24	80.00	28	93.33	22	75.86	21	63.64	**0.035**	95	77.87
Antidiabetic	14	46.67	21	70.00	11	37.93	15	45.45	0.075	61	50.00
Hypolipidemic	22	73.33	29	96.67	24	82.76	26	78.79	0.068	101	82.79
Antiplatelet	28	93.33	28	93.33	27	93.10	31	93.94	1.000	114	93.44
Anticoagulant	3	10.00	6	20.00	1	3.45	2	6.06	0.193	12	9.84
**ABI**									0.657		
Normal	4	13.33	7	23.33	5	17.24	5	15.15		21	17.21
Mild ischemia	6	20.00	2	6.67	9	31.03	8	24.24		25	20.49
Moderate ischemia	13	43.33	12	40.00	9	31.03	12	36.36		46	37.70
Severe ischemia	7	23.33	9	30.00	6	20.69	8	24.24		30	24.59
CWT *	355.00	287.50	300.00	330.00	500.00	220.00	300.00	300.00	0.225	375.00	300.00
**Severity of IC**									0.631		
Mild IC	16	53.33	14	46.67	20	68.97	16	48.48		66	54.10
Moderate IC	11	36.67	14	46.67	7	24.14	14	42.42		46	37.70
Severe IC	3	10.00	2	6.67	2	6.90	3	9.09		10	8.20
**Barthel questionnaire results**									0.134		
Mild dependence	1	3.33	4	13.33	2	6.90	7	21.21		14	11.48
Independence	29	96.67	26	86.67	27	93.10	26	78.79		108	88.52
VASCUQOL-6 questionnaire results *	20.50	3.75	19.00	7.00	22.00	4.00	19.50	3.00	**0.013**	20.00	4.25
SF-12: physical component score *	49.19	11.29	45.88	10.75	50.66	11.98	47.36	6.67	0.266	48.11	10.15
SF-12: mental component score *	59.31	13.82	60.05	13.95	60.70	7.54	58.83	13.25	0.649	59.81	13.96

* These numeric variables are described as median and inter-quartile range (IQR). Abbreviations: BMI, body mass index; COPD, chronic obstructive pulmonary disease; ABI, ankle-brachial index; CWT, claudication walk test; IC, intermittent claudication; SD, standard deviation.

**Table 2:** Adherence to physical exercise programs for intermittent claudication patients.

	Unsupervised Physical Exercise (USEP)		Supervised Physical Exercise (SEP)						
	Standard Advice (SA)		Walking Intervention (WI)		Strength Intervention (SI)		Concurrent Intervention (CI)		
	n = 30		n = 30		n = 29		n = 33		
	Median/n	IQR/%	Median/n	IQR/%	Median/n	IQR/%	Median/n	IQR/%	* p * -Value
**Ratio of adherence to** **physical exercise**	93.10	15.52	69.44	48.61	55.56	69.44	63.89	69.44	<0.001
**Adherence to** **physical exercise**	24	80.00%	13	43.33%	11	37.93%	15	45.45%	0.004
**Ratio of adherence to** **wearing a pedometer**	94.02	15.61	86.15	38.19	72.53	76.19	74.73	85.08	0.022
**Adherence to** **wearing a pedometer**	25	83.33%	17	56.67%	13	44.83%	16	48.48%	0.011

Abbreviations: IQR, interquartile range.

**Table 3:** Adherence to physical exercise programs for intermittent claudication patients of at least 50%.

	Standard Advice (SA)		Walking Intervention (WI)		Strength Intervention (SI)		Concurrent Intervention (CI)		
	n = 30		n = 30		n = 29		n = 33		
	n	%	n	%	n	%	n	%	* p * -Value
**Adherence to** **physical exercise ≥ 50%**	26	86.67%	20	66.67%	15	51.72%	20	60.61%	0.032
**Adherence to** **wearing a pedometer ≥ 50%**	27	90.00%	23	76.67%	17	58.62%	19	57.58%	0.014

**Table 4:** Relationship between adherence to physical exercise and ankle-brachial Index (ABI).

	Severe Ischemia			Moderate Ischemia			Mild Ischemia			Normal			
	ABI ≤ 0.50			ABI 0.51–0.70			ABI 0.71–0.89			ABI ≥ 0.90			
	n Global	n	%	n Global	n	%	n Global	n	%	n Global	n	%	* p * Value
**Adherence to physical exercise**													
Total	30	13	43.33	46	26	56.52	25	10	40.00	21	14	66.67	0.210
Standard advice	7	7	100.00	13	9	69.23	6	4	66.67	4	4	100.00	0.274
Walking intervention	9	3	33.33	12	7	58.33	2	0	0.00	7	3	42.86	0.550
Strength intervention	6	1	16.67	9	4	44.44	9	3	33.33	5	3	60.00	0.537
Concurrent intervention	8	2	25.00	12	6	50.00	8	3	37.50	5	4	80.00	0.285

Abbreviations: ABI, ankle-brachial index.

**Table 5:** Relationship between adherence to physical exercise and severity of intermittent claudication (IC).

	Severe + Moderate IC			Mild IC			
	Claudication Walk Test ≤ 300			Claudication Walk Test >300			
	n Global	n	%	n Global	n	%	* p * Value
**Adherence to physical exercise**							
Total	56	25	44.64	66	38	57.58	0.154
Standard advice	14	11	78.57	16	13	81.25	1.000
Walking intervention	16	7	43.75	14	6	42.86	0.961
Strength intervention	9	2	22.22	20	9	45.00	0.412
Concurrent intervention	17	5	29.41	16	10	62.50	0.056

Abbreviations: IC, intermittent claudication.

## Data Availability

No patient level data can be shared owing to local information governance and data protection regulations.

## Abbreviations

ICSPAD	Intermittent Claudication Symptomatic peripheral arterial disease
PAD	Peripheral artery disease
IC	intermittent claudication
CWT	Claudication Walk Test
SEP	supervised physical exercise program
USEP	unsupervised exercise program
WI	Walking intervention
SI	Strength intervention
CI	Concurrent intervention
SA	Standard advice
ABI	ankle-brachial index
BMI	body mass index
SD	standard deviations
IQR	interquartile range
